# Higher plasma transforming growth factor (TGF)-β is associated with kidney disease in older community dwelling adults

**DOI:** 10.1186/s12882-017-0509-6

**Published:** 2017-03-21

**Authors:** Tapan Mehta, Petra Buzkova, Jorge R. Kizer, Luc Djousse, Michel Chonchol, Kenneth J. Mukamal, Michael Shlipak, Joachim H. Ix, Diana Jalal

**Affiliations:** 1 University of Colorado Anschutz Medical Center, Aurora, USA; 2 University of Washington, Seattle, USA; 3 Albert Einstein College of Medicine, New York, USA; 4 Brigham and Women’s Hospital and Harvard Medical School, Boston, USA; 5 Beth Israel Deaconess Medical Center, Boston, USA; 6 University of California San Francisco School of Medicine, San Francisco, USA; 7 University of California, San Diego, USA; 8 Division of Renal Diseases and Hypertension, University of Colorado Anschutz Medical Campus, Campus Stop: C281, 12700 E. 19th Ave, Aurora, CO 80015 USA

**Keywords:** CKD, TGF-β, GFR, Older adults

## Abstract

**Background:**

TGF-β is induced in the vasculature with aging suggesting that high plasma TGF-β levels may be a risk factor for chronic kidney disease (CKD) in older adults.

**Methods:**

We conducted a cross-sectional analysis of the association between plasma TGF-β levels and CKD including data for 1722 older adults who had participated in the 1996/97 visit of the Cardiovascular Health Study (CHS). Prevalent CKD was defined as eGFR < 60 mL/min/1.73 m^2^ or urinary albumin/creatinine ratio (ACR) ≥30 mg/g. We also evaluated whether baseline TGF-β levels predicted change in eGFR, cardiovascular (CV) events, or mortality in longitudinal analysis.

**Results:**

Plasma TGF-β levels were significantly and independently associated with lower eGFR in cross-sectional analysis. Doubling of TGF-β was significantly associated with lower eGFR (β estimate after adjusting for CV risk factors = −1.18, 95% CI −2.03, −0.32). We observed no association with albuminuria. There was no association between baseline TGF-β and change in eGFR, but each doubling of TGF-β at baseline was associated with increased risk of a composite outcome of CV events and mortality, adjusted HR 1.10 (95% C.I. 1.02– 1.20, *p =* 0.006).

**Conclusion:**

In this large cohort of community-dwelling older individuals, high plasma TGF-β levels are modestly, but independently associated with lower eGFR but not with albuminuria in cross-sectional analysis. In addition, TGF-β levels are associated with increased risk of CV events and mortality. Further research is needed to determine the direction of association between plasma TGF-β and the risk of CKD and CKD-associated morbidities in older adults.

**Electronic supplementary material:**

The online version of this article (doi:10.1186/s12882-017-0509-6) contains supplementary material, which is available to authorized users.

## Background

In the United States, chronic kidney disease (CKD) prevalence is estimated to afflict approximately 11.5% of the adult population [1]. The pathophysiology of CKD differs depending on the primary cause of kidney injury. However, kidney disease progression, independent of the type of primary insult, occurs via a final common pathway of glomerulosclerosis and tubulointerstitial fibrosis [2]. The kidney responds to injury by releasing pro-inflammatory cytokines and growth factors such as transforming growth factor (TGF)-β. Sustained overexpression of TGF-β from continuous injury induces extracellular matrix accumulation in the diseased kidney [3] and ultimately leads to glomerular and tubulointerstitial fibrosis [4–6]. The role of TGF-β in kidney disease progression is further affirmed by data that administration of anti-TGF-β antibody attenuates fibrosis in different animal models of kidney injury indicating an important role of TGF-β in the fibrotic process [7–9]. Furthermore, several clinical studies have shown increased TGF-β expression in the kidneys of patients with glomerular disease including diabetic nephropathy [10, 11] and other inflammatory glomerulonephritides [12, 13].

Despite the established role of TGF-β in kidney disease progression in animal models, it remains unclear whether systemic TGF-β levels indicate kidney disease in humans. Some studies that have measured plasma levels of TGF-β in persons with diabetic kidney disease [14, 15] suggest that increased TGF-β levels predict progressive kidney disease in this patient population. In contrast to these findings, an analysis in the Chronic Renal Insufficiency Cohort study evaluated TGF-β levels in 3791 participants, almost half of whom had DM, and found no cross-sectional association between TGF-β levels and measures of CKD [16]. Thus, whether or not TGF-β levels are a risk factor for CKD in general remains uncertain.

CKD is more prevalent in the older population [1, 17], mainly owing to reduced eGFR rather than albuminuria [18]. Aging itself appears to associate with a higher prevalence of fibrotic kidneys [19]. The high prevalence of CKD in older adults is attributable not only to the presence of traditional risk factors such as diabetes and hypertension but may also be the result of age-related functional changes that occur in the kidney [20]. Importantly, epidemiological studies have identified arterial stiffness (which increases with age) as a predominant risk factor for progressive GFR decline in older people [21, 22]. We have previously shown TGF-β levels independently predict peripheral vascular disease in aged community-dwelling adults [23]. These findings are consistent with data that TGF-β is induced in the arterial wall with aging [24], such that apart from TGF-β production and its effects at the level of the nephron, upstream vascular generation could contribute to circulating TGF-β levels in older individuals. To our knowledge, no study has examined the potential association between plasma TGF-β levels and CKD in an older, community-living population. The Cardiovascular Health study (CHS) is a large observational cohort of community-dwelling adults aged ≥65 years that was designed to study traditional and novel risk factors for cardiovascular disease in older adults. Circulating TGF-β levels were measured on platelet-free plasma in a subset of CHS participants at the 1996/97 study visit in addition to estimated glomerular filtration rate (eGFR) and urinary albumin/creatinine ratio (ACR). Thus, we utilized cross-sectional data from CHS to test our hypothesis that higher plasma TGF-β levels associate with prevalent kidney disease in community-living older persons. We also evaluated whether TGF-β levels associated with longitudinal outcomes including change in eGFR, cardiovascular events, and mortality.

## Methods

### Study participants

The details of the study design, rationale, and study population of CHS have been described previously [25, 26]. Briefly, CHS is a community-based prospective cohort study designed to explore cardiovascular disease risk factors and stroke in individuals 65 years or older. The original cohort of the CHS consisted of 5201 participants who were randomly enrolled from the Medicare-eligibility lists in 4 US communities (Forsyth County, NC; Sacramento County, CA; Washington County, MD; and Allegheny County, PA) in 1989 to 1990. A supplemental cohort of 687 mostly African-American participants was recruited in 1992–1993.

The eligible individuals were aged ≥65 years, not institutionalized, expected to remain in the current community for ≥3 years, not under active cancer treatment, and able to give written informed consent. Follow-up examinations were done at annual visits through 1998/99 and again in 2006/07. Interviews were also conducted through biannual telephone calls. The study was approved by the institutional review boards of the 4 clinical sites including: University of California, Davis (Sacramento County, Sacramento, CA), Johns Hopkins University (Washington County, Hagerstown, MD), Wake Forest University School of Medicine (Forsyth County, Winston-Salem, NC), University of Pittsburgh (Pittsburgh, PA) [27]. All participants gave written informed consent including the use of de-identified samples and data for future analyses.

### Measurement of TGF-β

TGF-β1 was measured in 2011–2012 on EDTA-stored plasma samples from the 1996/97 visit by ELISA (Quantikine Human TGF-β1 Immunoassay; R&D Systems, Minneapolis, MN). Inter- and intra-assay coefficients of variation were between 1.9 and 2.9% and 6.4% to 9.3%, respectively. Platelets are a major source of TGF-β as a result of platelet degranulation, such that if the plasma is not platelet poor, platelet contamination in plasma samples can artificially increase levels of TGF-β [28]. Presumed platelet contamination of the plasma resulting in elevated levels of TGF-β was found in pilot studies at two of the study sites. Therefore, TGF-β was only measured *a priori* in 1722 participants at the two remaining sites and our final analysis included these individuals. Of the two sites at which TGF-β was not measured, one site (Washington County, Maryland) did not enroll an African American cohort in 1992–1993, leading to modest differences in other participant characteristics across clinic sites.

### Kidney function measurements

Blood samples were collected after an overnight fast and stored at −70 °C using standardized protocols at the 1996/97 visit for the baseline measurements and the 2006/07 for the longitudinal analysis of kidney disease progression. The primary outcome of interest was kidney function estimated by serum cystatin C and albuminuria. Cystatin C was measured using a particle-enhanced immunonephelometric assay with a BN II nephelometer on plasma specimens (N Latex Cystatin C; Dade Behring (now Siemens Health-care Diagnostics Inc.), Deerfield, IL, USA) [29]. The assay range was 0.195–7.330 mg/L, with the reference range for young, healthy individuals reported as 0.53–0.95 mg/L. For cystatin C, intra-assay coefficients of variation (CVs) range from 2.0 to 2.8% and inter-assay CVs range from 2.3 to 3.1%. Glomerular filtration rate (GFR) was calculated using the CKD-EPI cystatin C equation [30]. GFR expressed as mL/min per 1.73 m^2^ of body surface area, and serum cystatin C expressed in mg/L. In categorical analyses, CKD was defined as eGFR <60 mL/min/1.73 m^2^. In addition, in the longitudinal analysis we evaluated change in eGFR from the 1996/97 visit to the 2006/07 visit as an outcome since the measurements of kidney function were repeated in a subset of survivors at this time. Albuminuria was assessed using urinary ACR calculated from spot urinary albumin and creatinine levels. Albuminuria was defined as ACR ≥30 mg/g in categorical analyses.

### Cardiovascular disease and mortality

CHS has previously reported on the association between plasma TGF-β levels and cardiovascular disease, including heart failure, myocardial infarction, and stroke through 2010 [31]. It had similarly reported on the association between TGF-β and mortality in another manuscript [32]. Here, we report on the association between plasma TGF-β levels and cardiovascular events and mortality in longitudinal analysis considering the longer duration of follow-up available since our previous publications (2014) [31, 32]. Participants were followed from the baseline visit (1996/97 for this analysis) until death, loss to follow-up, and the end of follow up on December 31^st^, 2014. Cardiovascular events (including heart failure, myocardial infarction, and stroke) and death were adjudicated by the CHS outcome-assessment committee on the basis of patient reports, physician diagnoses, medical records, and medication use [33, 34].

### Covariate assessment

We used covariate measurement from the 1996/97 visit. Age, sex, race, and smoking history variables were collected by self-report. Smoking history was determined by questionnaire and categorized as current, former, or never. Blood pressure (BP) was measured twice in seated position after 5 min rest using standard mercury sphygmomanometer. Hypertension was defined by average seated systolic blood pressure ≥140 mmHg, diastolic blood pressure ≥90 mmHg, or by the current use of antihypertensive medications. We defined diabetes mellitus by use of oral hypoglycemic agents or insulin, or as a fasting glucose level ≥126 mg/dL. Height (m) and weight (kg) were measured to calculate body mass index (BMI: kg/m^2^). Low density lipoprotein (LDL)-cholesterol and triglycerides were measured during CHS year 5 (1992–1993) visit. Serum triglyceride levels were measured with the Olympus Demand System (Olympus, Lake Success, NY, USA); LDL-cholesterol concentrations were calculated by the Friedewald equation [35]. C-reactive protein (CRP) was measured by an enzyme linked immunosorbent (ELISA) assay. Prevalent cardiovascular disease (CVD) was defined by history of myocardial infarction, angina, or stroke.

### Statistical analysis

Clinical characteristics are presented overall and by quartiles of plasma TGF-β. Continuous data are presented as mean ± standard deviation (SD), and categorical variables as counts (%). Variables with skewed distribution are shown as median (inter quartile range, IQR). Covariates were compared across plasma TGF-β quartiles using a linear trend test, and chi-square test for categorical variables. TGF-β was skewed, so in continuous variable analyses it was log base 2-transformed so that the β estimates can be interpreted as per doubling of TGF-β. Correlations were assessed with Pearson correlation coefficient. To evaluate whether plasma levels of TGF-β are associated with kidney function we first conducted cross-sectional linear regression analysis with plasma TGF-β as the predictor variable and kidney function measures (eGFR and log2 ACR) as the outcome variables. To examine whether TGF-β levels correlated with clinically significant CKD, we conducted logistic regression analysis for TGF-β and estimated GFR <60 mL/min/1.73 m^2^, and subsequently for TGF-β and ACR ≥30 mg/g. Considering the potential bias of eGFR equations in the diagnosis of CKD between eGFR 45–59 mL/min/1.73 m^2^, we also evaluated the association between TGF-β levels and CKD defined as eGFR <45 mL/min/1.73 m^2^ [36]. Several models were applied for multivariate adjustment. M1 adjusted for age, gender, and black race. M2 adjusted for the covariates in M1 as well as BMI, hypertension, DM, smoking status, LDL-cholesterol, triglycerides, previous history of CVD, and log2 CRP. Additionally, we adjusted for estimated GFR together with the covariates included in M2 (when evaluating the association with ACR) or ACR (when evaluating the association with eGFR). To evaluate whether baseline TGF-β predicted kidney disease progression, we conducted a similar analysis longitudinally. Only a subset of survivors (*n =* 478) had cystatin C measurements at the 2006/07 visit. Considering this small number, we were unable to evaluate whether TGF-β associated with incident CKD and the designated outcome included change in CKD-EPI eGFR (from the 1996/97 visit to the 2006/07 visit). Cox proportional hazard models were utilized to evaluate whether baseline TGF-β levels predicted cardiovascular (CV) events, death, or a composite outcome of CV events and death. The same covariates included in the linear regression models were included in the cox proportional hazard models. For all statistical tests, a two-tailed P-value < 0.05 was considered statistically significant. We did not adjust for multiple testing. Analyses were conducted using R (R Development Core Team (2015)) [37].

## Results

### Clinical characteristics of the participants

TGF-β levels were available for 1722 (39%) individuals out of a total cohort of 4413 at CHS year 9 (1996/97) visit. ACR values were available for 1541 (89.5%) of participants with TGF-β levels. Participants with TGF-β measurements differed from those without available measures in several ways. They were younger, more frequently male and black, and had lower blood pressure. In addition, as shown in Additional file 1: Table S1, they less often had prevalent CVD. Among those with available TGF-β levels, the median (IQR) of TGF-β levels was 3482 (2042–6153) ng/L.

The participant characteristics by TGF-β quartiles at baseline are shown in Table 1. Compared to participants in the lower TGF-β quartiles, those with TGF-β levels in the highest quartile were more frequently black, were more likely to have DM, to have higher CRP, and to be current smokers. Age and previous history of CVD were not significantly different according to TGF-β quartiles. eGFR was significantly lower in quartiles 2, 3, and 4 compared to quartile 1. Albuminuria did not differ significantly among the groups. Of note, median TGF-β was 3482 (IQR 2042–6153) for all the participants. Median TGF-β was higher for those with eGFR <60 ml/min/1.73 m^2^ compared to those with eGFR >60 ml/min/1.73 m^2^ (3747 [IQR 2278–6431] *vs.* 3377 [IQR 1962–6075] ng/L), with p value = 0.05 (based on Mood’s test of medians).

**Table 1:** Baseline characteristics of individuals according to plasma TGF-β quartiles

Variables	Total *n =* 1722	Quartile 1 *n =* 431	Quartile 2 *n =* 430	Quartile 3 *n =* 430	Quartile 4 *n =* 431	*P-*value
Age (years)	78 ± 5	78 ± 4	78 ± 5	78 ± 5	78 ± 5	0.22
Gender (male %)	40	38	41	43	38	0.27
Black race (%)	22	20	18	20	31	<0.01
Current smoker	9	11	6.0	9	11	0.01
Diabetes mellitus (%)	16	13	13	17	20	<0.01
Hypertension (%)	59	62	57	56	63	0.14
Prevalent CVD (%)	24	23	24	25	23	0.89
BMI (kg/m^2^)	27 ± 5	27 ± 5	27 ± 5	27 ± 5	27 ± 4	0.05
CRP (mg/L)^b^	2.43 (1.12–5.41)	2.18 (1.07–4.84)	2.47 (1.13–4.97)	2.38 (1.09–5.06)	2.78 (1.28–7.43)	0.14
LDL-cholesterol (mg/dL)	83 ± 87	81 ± 86	83 ± 87	87 ± 90	83 ± 88	0.68
Triglycerides (mg/dL)	138 ± 78	136 ± 73	139 ± 76	134 ± 78	143 ± 84	0.43
eGFR (ml/min/1.73 m^2^)	71 ± 20	75 ± 19	70 ± 20	70 ± 19	70 ± 20	<0.01
ACR (mg/g)^a^	9.3 (5–20.2)	8.6 (4.9–16)	9.3 (5.4–20.5)	9.65 (4.9–20.55)	9.5 (4.8–22.35)	0.46
% with eGFR <60 ml/min/1.73 m^2^	25	20	27	28	27	0.01
% with ACR ≥30 mg/g	18	15	19	18	21	0.23

Values are expressed as means ± standard deviation or (%) = percent; *BMI* body Mass Index, *GFR* glomerular filtration rate, *ACR* albumin/creatinine ratio, *CRP* C-reactive protein, *CVD* cardiovascular disease. Prevalent CVD was defined by history of myocardial infarction, angina, or stroke

^a^, ^b^values expressed as median (Interquartile range). *P*-values are from a linear trend test across quartiles for continuous variables and from chi^2^ test for binary and categorical variables

### The cross-sectional association between TGF-β levels and eGFR or ACR as continuous variables

**Fig. 1:**
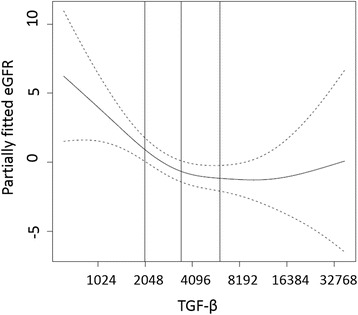
This illustrates the partially-fitted estimated GFR (eGFR) when using splines for TGF-β levels in Generalized Additive Models (GAM) adjusting for age, sex, black race, smoking, hypertension, diabetes mellitus, body mass index, LDL-cholesterol, triglycerides, history of cardiovascular disease, and C-reactive protein. The dotted lines are the 95% confidence bands. The x axis is on log2 scale and the three vertical lines show Q1, median, and Q3 of TGF-β

Linear regression analysis revealed that each doubling of TGF-β was significantly associated with lower eGFR (β estimate = −1.54; 95% C.I. -2.44, −0.63; *p <* 0.001), although this was marginally non-significant with respect to log2 ACR (β estimate = 0.09; 95% C.I. 0, 0.18; *p =* 0.055) in unadjusted analysis. As shown in Table 2, the association TGF-β and eGFR was slightly attenuated but remained statistically significant after adjusting for demographics in model 1 (β estimate = −1.41, 95% CI −2.27, −0.55) and including cardiovascular risk factors in model 2 (β = −1.18, 95% CI −2.03, −0.32). Additional adjustment for ACR yielded a β estimate of −1.07 (95% CI −1.95, −0.19). Further inclusion of congestive heart failure and claudication in the model had no influence on the association between TGF-β and eGFR. In contrast, the association between TGF-β and ACR was not significant after adjustment of demographics or in any of the other multivariable models. Since the generalized additive model in relation to eGFR showed that the association tended to plateau above the median value of TGF-β (Fig. 1 log2 TGF-β corresponds to approximately 4096 ng/L) we also evaluated the association for quartiles of the biomarker.

**Table 2:** Linear regression analysis of the association between plasma TGF-β (per doubling) and eGFR and ACR

Model	eGFR		Urinary ACR	
	β (95% C.I.)	*p-*value	β (95% C.I.)	*p-*value
M0	–1.54 (–2.44,–0.63)	<0.001	0.09 (0,0.18)	0.055
M1	–1.41 (–2.27,–0.55)	<0.001	0.06 (–0.03,0.15)	0.19
M2	–1.18 (–2.03,–0.32)	0.007	0.04 (–0.05,013)	0.35

M0: unadjusted analysis

M1: M0 + age, male gender, and black race

M2: M1 + current smoking status, hypertension, diabetes mellitus, body mass index, LDL-cholesterol, triglycerides, history of cardiovascular disease, and C-reactive protein

When TGF-β levels were modeled as quartiles, we found that TGF-β levels in quartiles 2, 3, and 4 were significantly associated with lower eGFR relative to the first quartile. In the unadjusted analysis eGFR was lower by 4.55 (95% C.I. -7.14, −1.95), 4.92 (95% C.I. -7.52, −2.32), and 4.47 (95% C.I. -7.07, −1.88) ml/min/1.73 m^2^ for quartiles 2, 3, and 4 respectively. These statistically significant associations were slightly attenuated but remained significant after full adjustment (Table 3). We did not find a significant association between higher TGF-β levels and urine ACR in any unadjusted or adjusted analyses.

**Table 3:** Linear regression analysis of the association between TGF-β quartiles and eGFR and urinary ACR

	Quartile 1 β estimate (95% CI)	Quartile 2 β estimate (95% CI)	Quartile 3 β estimate (95% CI)	Quartile 4 β estimate (95% CI)
eGFR				
M0	REF	–4.55 (–7.14,–1.95)	–4.92 (–7.52,–2.32)	–4.47 (–7.07,–1.88)
M1	REF	–3.8 (–6.26,–1.34)	–4.02 (–6.48,–1.56)	–4.36 (–6.83,–1.89)
M2	REF	–4.57 (–7,–2.14)	–4.68 (–7.09,–2.27)	–3.86 (–6.28,–1.44)
Urinary ACR				
M0	REF	0.24 (–0.02,0.5)	0.21 (–0.06,0.47)	0.25 (–0.01,0.51)
M1	REF	0.21 (–0.05,0.47)	0.16 (–0.1,0.42)	0.18 (–0.08,0.44)
M2	REF	0.25 (0,0.5)	0.20 (–0.05,0.45)	0.15 (–0.1,0.4)

M0: unadjusted analysis

M1: M0 + age, male gender, and black race

M2: M1 + current smoking status, hypertension, diabetes mellitus, body mass index, LDL-cholesterol, triglycerides, history of cardiovascular disease, and C-reactive protein

### The cross-sectional association between TGF-β levels and clinically-significant CKD

**Fig. 2:**
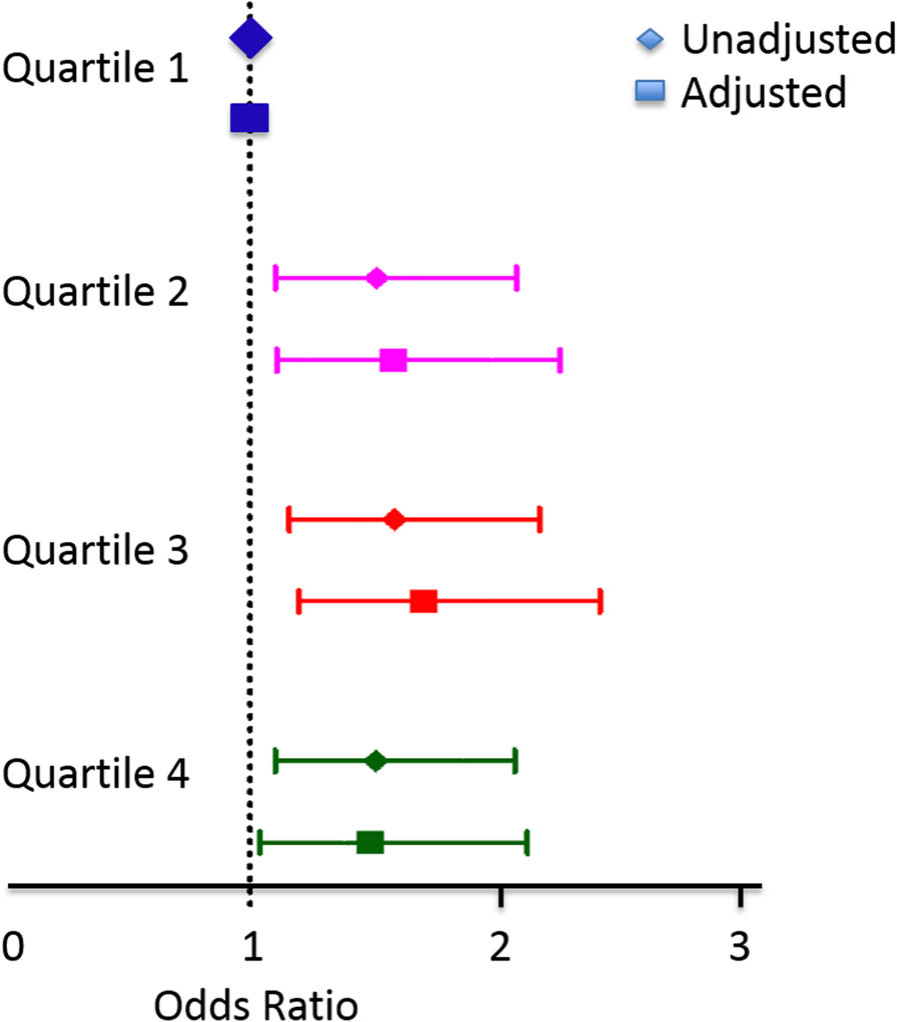
Cross-sectional association between TGF-β quartiles and CKD defined as eGFR <60 mL/min/1.73 m^2^. Quartiles 2, 3, and 4 were compared to quartile 1. The shown adjusted odds ratio are adjusted for age, sex, black race, smoking, hypertension, diabetes mellitus, body mass index, LDL-cholesterol, triglycerides, history of cardiovascular disease, and C-reactive protein: Quartile 2: OR for CKD was 1.59 (95% C.I. 1.11, 2.28; *p* value = 0.01) Quartile 3: OR for CKD was 1.72 (95% C.I. 1.20, 2.45; *p* value = 0.003). Quartile 4: OR for CKD was 1.50 (95% C.I. 1.04, 2.15; *p* value = 0.03)

To determine whether TGF-β levels associated with clinically significant CKD, we evaluated the binary end point of either eGFR <60 ml/min/1.73 m^2^ or with ACR ≥30 mg/g in logistic regression analysis. As depicted in Fig. 2, TGF-β levels in quartiles 2, 3, and 4 were significantly associated with CKD defined as eGFR <60 ml/min/1.73 m^2^. In unadjusted analysis, compared to the participants in quartile 1 of TGF-β levels, participants in quartile 2 had 53% higher odds of eGFR <60 ml/min/1.73 m^2^ (95% C.I. 1.11, 2.1), participants in quartile 3 had 60% higher odds of eGFR <60 ml/min/1.73 m^2^ (95% C.I. 1.16, 2.2), and participants in quartile 4 had 52% higher odds of eGFR <60 ml/min/1.73 m^2^ (95% C.I. 1.11, 2.10). This remained significant for quartiles 2, 3, and 4 after adjusting for demographics and cardiovascular risk factors with OR of 1.59 (95% C.I. 1.11, 2.28), 1.72 (95% C.I. 1.20, 2.45), and 1.50 (95% C.I. 1.04, 2.15), respectively. Of note, when CKD was defined as eGFR < 45 ml/min/1.73 m^2^ [36], the fully-adjusted ORs for CKD were 3.59 (95% C.I. 1.66, 7.77; *p <* 0.001), 4.58 (95% C.I. 2.16, 9.68; *p <* 0.001), and 4.24 (95% C.I. 2.0, 9.02; *p <* 0.001) for quartiles 2, 3, and 4 compared to quartile 1. There was no consistent evidence of an association between TGF-β quartiles and ACR ≥30 mg/g. Quartile 4 of TGF-β levels was associated with 34% higher odds of having ACR ≥30 mg/g compared to quartile 1 in unadjusted analysis but this was no longer statistically significant after adjusting for demographic and cardiovascular risk factors (OR = 1.34, 95% C.I. 0.89, 2.04, *p =* 0.17).

### The association between baseline TGF-β levels and longitudinal outcomes

To evaluate whether TGF-β levels predicted kidney disease progression, we evaluated whether doubling of TGF-β associated with change in eGFR. We found no association between TGF-β levels at baseline and change in CKD-EPI eGFR between the years 1996/97 and 2006/07 in unadjusted or adjusted models. Only 478 individuals had cystatin C measurements at the 2006/07 visit.

Between the 1996/97 visit and the end of follow-up in 2014, 409 out of the studied 1722 participants died with a median follow up of 10.3 years (IQR 5.7–15.8). Of the surviving 1312 participants, 411 were reported to have a CV event and the median follow up was 9.5 years (IQR 5.1-14.5 years). Similar to the previous report [32], doubling of baseline TGF-β was associated with an increased risk of death. In adjusted analysis, the hazard ratio (HR) for death was 1.10 (95% C.I. 1.02, 1.13, *p =* 0.008). This was somewhat attenuated to a HR of 1.10 (95% C.I. 1.01, 1.10, *p =* 0.019) and 1.10 (95% C.I. 1.0, 1.12, *p =* 0.05) for model 1 and model 2, respectively. Also similar to the previous report [31], we found no association between baseline TGF-β levels and CV events in unadjusted analysis (HR was 1.10, 95% C.I. 0.99, 1.20, *p =* 0.074). After adjusting for the covariates included in model 2, doubling of TGF-β carried a HR of 1.10 for CV events (95% C.I. 1.0–1.22, *p =* 0.05). Doubling of baseline TGF-β was associated with higher risk of the composite outcome of CV events or death. The unadjusted HR for the composite outcome 1.10 (95% C.I. 1.02, 1.20, *p =* 0.005). This association remained significant with HR of 1.10 (95% C.I. 1.03, 1.20, *p =* 0.004) and 1.10 (95% C.I. 1.02, 1.20, *p =* 0.006) for model 1 and model 2, respectively.

## Discussion

In this cross-sectional analysis of community-living older adults, higher levels of circulating TGF-β were associated with both lower eGFR as a continuous variable, and with CKD defined as eGFR <60 mL/min/1.73 m^2^. We found no association between TGF-β levels and albuminuria. The associations between TGF-β and eGFR were independent of demographic and cardiovascular risk factors, C-reactive protein, and albuminuria. In addition, the association between TGF-β levels and eGFR was similar in quartiles 2, 3, and 4 when compared to quartile 1, suggesting that plasma TGF-β above a threshold may be a risk factor for CKD in older adults. The association between TGF-β levels and clinically-significant CKD persisted even when CKD was defined as <45 mL/min/1.73 m^2^ suggesting that higher TGF-β levels are indicative of CKD, not merely an aging kidney [36]. Thus, we provide new insights to biology associated with CKD in older community dwelling adults, and find that measurement of circulating TGF-β may give insights to kidney disease in this setting. We were not able to find an association between baseline TGF-β levels and change in eGFR between the 1996/97 and the 2006/07 visits. This is likely due to small number of individuals with available kidney function measurement as well as the competing outcomes of CV events and mortality as TGF-β levels were associated with the composite outcome of CV events and mortality over an extended period of follow up.

TGF-β is pleiotropic cytokine involved in kidney disease progression as in vivo experimental studies have shown renal TGF-β overproduction by mesangial cells [38], tubular epithelial cells [39], interstitial fibroblasts, and macrophages [40, 41]. Induction of TGF-β has been shown to cause extracellular matrix accumulation in the glomeruli and interstitium [3] leading to progressive kidney disease [4–6]. Consistently, inhibition of TGF-β via anti-TGF-β antibody has been shown to attenuate fibrosis in animal models of kidney disease [7–9]. TGF-β expression has been demonstrated in the kidneys of individuals with glomerular disease such as diabetic nephropathy [10, 11], focal segmental glomerulosclerosis secondary to human immunodeficiency virus (HIV) infection [12], and other glomerulonephritides such as IgA nephropathy, and lupus [13]. However, no study to date has examined whether TGF-β levels are elevated in older adults, a population at high risk for CKD.

Plasma levels of TGF-β have been measured in 2 studies in subjects with diabetic kidney disease. Wong et el. measured plasma TGF-β levels in participants of the Action in Diabetes and Vascular Disease: Preterax and Diamicron MR Controlled Evaluation (ADVANCE) trial. (The study compared the effect of perindopril and indapamide on macrovascular and microvascular outcomes in patients with type 2 DM) [14]. In a post-hoc analysis they identified 102 participants with progressive diabetic kidney disease over a period of 5 years and compared them to 179 participants whose kidney disease did not progress. Kidney disease progression was defined as doubling of serum creatinine, need for renal replacement therapy, or death due to renal disease during the 5-year follow up period. They found that baseline TGF-β levels were higher in participants with progressive kidney disease compared to the participants whose kidney disease did not progress. The association between baseline TGF-β levels and kidney disease progression here was independent of baseline eGFR and albuminuria. In the other study of subjects with diabetic nephropathy Sharma et al. measured TGF-β levels in participants randomized to placebo (*n =* 24) or captopril (*n =* 34). Here kidney disease progression, defined as loss of GFR, was slower in those whose TGF-β levels were reduced over a period of 6 months [15]. Collectively, these data suggest that higher TGF-β levels are associated with progressive diabetic nephropathy.

In contrast to the aforementioned studies, a recent cross-sectional analysis by Gupta et al. evaluated TGF-β levels in 3791 of the Chronic Renal Insufficiency Cohort (CRIC) participants, almost half of whom had DM. The investigators found no correlation between TGF-β levels and measures of kidney function including eGFR or urinary ACR [16]. Compared to the participants included in our analysis, participants in CRIC were younger, had a higher prevalence of HTN and DM, in addition to lower eGFR. It is possible that circulating TGF-β is a more sensitive marker of CKD in older adults who have a higher prevalence of arterial stiffness. This is consistent with the role arterial stiffness plays in CKD in the elderly [21] and considering that TGF-β is induced in the arterial wall with aging [24].

We found no association between plasma levels of TGF-β and albuminuria in our population of older adults. TGF-β is known to affect the glomerular basement membrane in several ways that lead to increased proteinuria such as induction of podocyte and endothelial to mesenchymal transition and glomerular basement membrane thickening [42]. Based on this, we expected that increased TGF-β levels would associate with albuminuria. It is possible, however, that increased TGF-β levels in our study are the result of induced production in tissues other than the kidney such as the vasculature. As such, systemic levels of TGF-β may not accurately reflect TGF-β production in the kidney. This is consistent with our previous work that TGF-β levels are associated with peripheral vascular disease [23] and with our findings in this analysis that TGF-β levels are associated with the composite end point of CV events/mortality.

There are several limitations to our study. First, the association between TGF-β and eGFR is cross-sectional so we are unable to draw conclusions on the direction of associations. Although it is unknown whether TGF-β is filtered by the kidney in humans animal data suggest that TGF-β is predominantly cleared by the liver [43]. In addition, although some studies have described forms of TGF-β with small molecular weight (for example 12.5, 25, 50, and 90 kDa in breast cancer tissue [44]), in humans, TGF-β is known to be synthesized and secreted in a biologically latent form as a high-molecular weight complex (135 kD) that is highly unlikely to be filtered in the absence of proteinuric glomerular disease [45, 46]. Thus, while our data cannot determine whether high TGF-β is associated with future decline in kidney function or if low eGFR raises TGF-β, we hypothesize the former association on the basis of these prior studies. Future studies with longitudinal data are an important next step in this area of research. Second, plasma levels of TGF-β were measured at a single time point on stored samples. It is unclear whether TGF-β is stable over an extended period of time and it is unknown whether plasma TGF-β levels exhibit inter and intra-subject variability over time, or whether intra-individual changes over time are associated with longitudinal changes in kidney function. In addition, we cannot guarantee the complete absence of platelet contamination at the sites included in this analysis, although we believe this is unlikely since we conducted pilot studies at all sites to evaluate this. Of note, we identified potential platelet contamination at 2 sites and excluded all the samples collected at both sites. Finally, because of the observational nature of the study, we cannot eliminate the possibility of residual confounding by imprecisely measured risk factors or unmeasured risk factors. Notwithstanding these weaknesses, the study has several strengths including the consistency of TGF-β measurement across samples and the large number of participants from a community-based cohort.

## Conclusions

In this analysis, we show for the first time that higher levels of plasma TGF-β are independently associated with lower eGFR and higher prevalence of CKD among community-dwelling older adults. We observed no statistically significant association of TGF-β with albuminuria. The lack of an association with GFR decline in our population is likely a reflection of the small number of participants with follow up kidney function measurements and the competing risk of other outcomes such as CV events and mortality. Our findings suggest that increased TGF-β may be a marker of vascular disease that contributes to GFR decline, CV events, and mortality in the aged. Future longitudinal studies are needed to assess whether circulating TGF-β levels are associated with more rapid loss of kidney function over time in community-living older adults, and whether therapies that lower TGF-β and fibrosis may play a role in preventing or treating CKD and CKD-associated co-morbidities in older adults.

## Additional file

Additional file 1: Table S1. Clinical characteristics by availability of TGF-β levels. (DOCX 13 kb)

## Abbreviations

ACR: Albumin/creatinine ratio
BMI: Body mass index
BP: Blood pressure
CHS: Cardiovascular Health study
CKD: Chronic kidney disease
CRP: C-reactive protein
CVD: Cardiovascular disease
DM: Diabetes mellitus
eGFR: Estimated glomerular filtration rate
IQR: Interquartile range
LDL: Low density lipoprotein
SD: Standard deviation
TGF-β: Transforming growth factor-β
