# Supplementary material for: Higher plasma transforming growth factor (TGF)-β is associated with kidney disease in older community dwelling adults
Source: BMC Nephrol. 2017 Mar 21;18:98. doi: 10.1186/s12882-017-0509-6 (PMC5359982; doi:10.1186/s12882-017-0509-6)
Supplement: Additional file 1: Table S1. — Clinical characteristics by availability of TGF-β levels. (DOCX 13 kb) [file 12882_2017_509_MOESM1_ESM.docx]

**Supplementary Table 1: Clinical characteristics by availability of TGF-β levels**

| **Variables** | **TGF-β levels unavailable**  **n=2691** | **TGF-β levels available**  **n=1722** | **P-value** |
| --- | --- | --- | --- |
| **Age (years)** | 79±5 | 78±5 | <0.01 |
| **Gender (male %)** | 37.2 | 40.2 | 0.05 |
| **Black race (%)** | 13.2 | 22.2 | <0.01 |
| **Current smoker (%)** | 6.7 | 9.1 | 0.01 |
| **Diabetes mellitus (%)** | 18.7 | 15.6 | 0.02 |
| **Hypertension (%)** | 69.5 | 59.4 | <0.01 |
| **Prevalent CVD (%)** | 28.1 | 23.8 | <0.01 |
| **CRP (mg/L)** | 4.6±7.7 | 4.9±8.3 | 0.34 |
| **LDL- cholesterol (mg/dL)** | 74±85 | 83±87 | <0.01 |
| **Triglycerides (mg/dL)** | 148±92 | 138±78 | <0.01 |
| **eGFR**  **(ml/min/1.73m^2^)** | 70±19 | 71±20 | <0.01 |
| **ACR (mg/g)** | 73±321 | 61±353 | 0.3 |
| **% with eGFR < 60 ml/min/1.73m^2^** | 29 | 25.3 | 0.02 |
| **% with ACR ≥ 30 mg/g** | 23.6 | 18.4 | <0.01 |

Values are expressed as means ± standard deviation or (%) = percent; BMI = body Mass Index; GFR = glomerular filtration rate, ACR= albumin/creatinine ratio, CRP=C-reactive protein, CVD = cardiovascular disease. P-values are from a linear trend test across quartiles for continuous variables and from chi^2^ test for binary and categorical variables.
